# Adverse Outcome of Early Recurrent Ischemic Stroke Secondary to Atrial Fibrillation after Repeated Systemic Thrombolysis

**DOI:** 10.1155/2013/371642

**Published:** 2013-08-06

**Authors:** Luciano A. Sposato, Valeria Salutto, Diego E. Beratti, Paula Monti, Patricia M. Riccio, Claudio Mazia

**Affiliations:** ^1^Vascular Research Institute, INECO Foundation, Pacheco de Melo 1860, Ciudad de Buenos Aires (C1126AAB), Buenos Aires, Argentina; ^2^Department of Neurology, Alfredo Lanari Institute of Medical Investigations, University of Buenos Aires, Buenos Aires, Argentina; ^3^Department of Medicine, Alfredo Lanari Institute of Medical Investigations, University of Buenos Aires, Ciudad de Buenos Aires, Argentina

## Abstract

*Background*. Recurrent ischemic stroke is associated with adverse neurological outcome in patients with atrial fibrillation. There is very scarce information regarding the neurological outcome of atrial fibrillation patients undergoing repeated systemic thrombolysis after early recurrent ischemic stroke. *Clinical Case and Discussion*. We describe a case of a 76-year-old woman with known paroxysmal atrial fibrillation who was admitted because of an acute right middle cerebral artery ischemic stroke and who underwent repeated systemic thrombolysis within 110 hours. The patient underwent systemic thrombolysis after the first ischemic stroke with almost complete neurological recovery. On the fourth day after treatment, an acute left middle cerebral artery ischemic stroke was diagnosed and she was treated with full-dose intravenous recombinant tissue plasminogen activator. A hemorrhagic transformation of the left middle cerebral artery infarction was noted on follow-up cranial computed tomographic scans. The patient did not recover from the second cerebrovascular event and died 25 days after admission. *Conclusion*. To the best of our knowledge, this is the second case reporting the adverse neurological outcome of a patient with diagnosis of atrial fibrillation undergoing repeated systemic thrombolysis after early recurrent ischemic stroke. Our report represents a contribution to the scarce available evidence suggesting that repeated systemic thrombolysis for recurrent ischemic stroke should be avoided.

## 1. Introduction

There is strong evidence that treatment of acute ischemic stroke with intravenous (IV) recombinant tissue plasminogen activator (rtPA) reduces long-term disability [1]. However, because of the risk of major bleeding, there is a need for careful selection of potentially treatable patients. Eligibility standards are based on inclusion and exclusion criteria used in large randomized clinical trials [2–4]. Among several contraindications, IV rtPA should not be used in patients who had suffered an ischemic stroke within the previous 3 months [1]. The repeated use of IV rtPA in the 3-month window may be associated to a higher risk of cerebral bleeding and to potential anaphylactic reactions [5]. There is scarce information about the repeated use of IV rtPA for early recurrent ischemic stroke [6–9], and serious concerns have been raised regarding this matter [10].

We report a case of repeated used of IV rtPA within 110 hours in a patient with early recurrent ischemic stroke associated to atrial fibrillation, and we discuss its pathophysiological, clinical, and prognostic implications.

## 2. Clinical Case and Discussion

**Figure 1:**
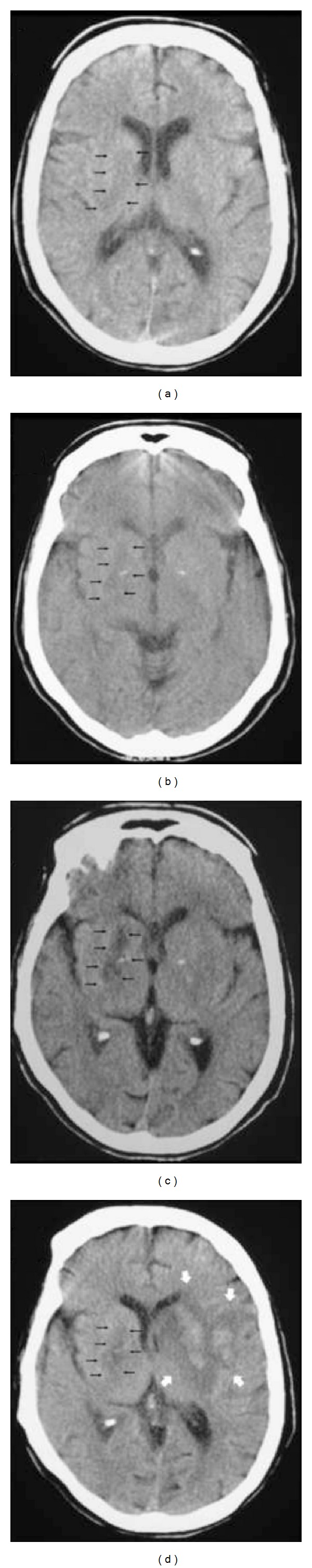
Cranial CT scan performed 100 minutes after first ischemic stroke onset (Panel a) and 24 hours after first IV thrombolysis (Panel b) shows a right lenticular hypodensity compatible with an acute cerebral infarction (black arrows). Cranial CT scan performed 30 minutes after second ischemic stroke onset (Panel c) and 48 hours after second systemic thrombolysis (Panel d) shows a subacute right lenticular infarction (black arrows) and a new predominantly subcortical ischemic lesion in the anterior region of the left middle cerebral artery territory with a PH2 hemorrhage (white arrows).

A 76-year-old woman was admitted to the Instituto de Investigaciones Médicas “Alfredo Lanari” because of left-sided numbness and slurred speech. On neurologic examination, she was not alert but arousable by minor stimulation. Examination of visual fields revealed a left homonymous hemianopia. A total paralysis of the left lower face was noted, and no movement could be elicited on the left arm and left leg. A left hemisensory deficit, moderate dysarthria, and profound left hemi-inattention were also evidenced. National Institutes of Health Stroke Scale (NIHSS) score was 18 points. Laboratory results were unremarkable. Head computed tomography (CT) scan performed 100 minutes after symptoms onset showed a subtle hypodensity of the right lenticular nucleus (Figure 1(a)). Electrocardiogram showed sinus rhythm.

The patient had a history hypertension, paroxysmal atrial fibrillation, hyperlipidemia, dilated cardiomyopathy, two acute myocardial infarctions, congestive heart failure, and pulmonary hypertension. She had undergone coronary artery stenting after the second myocardial infarction, and an implantable cardioverter-defibrillator was placed 6 years after. She was taking acetylsalicylic acid (100 mg/day), spironolactone (25 mg/day), furosemide (20 mg/day), carvedilol (6,125 mg BID), and simvastatin (20 mg/day). 

IV rtPA (0.9 mg/kg) was started 120 minutes after stroke onset. After 24 hours, there was a significant neurological improvement (NIHSS score of 3). The patient persisted with total lower left facial palsy and mild left leg paresis, and the head CT scan showed the same right lenticular nucleus hypodensity (Figure 1(b)) compatible with an acute cerebral infarction.

Carotid Doppler ultrasound revealed mild bilateral atherosclerotic disease with less than 10% stenoses and transesophageal echocardiography showed left ventricular ejection fraction of 31% and an atherosclerotic debris of the aortic arch. Anticoagulation with low-molecular-weight heparin was initiated 24 hours after IV rtPA. 

On the fourth day after treatment, a sudden neurological worsening was noted. The patient was alert but did not respond to simple commands. A conjugate deviation of the eyes that could not be overcome by voluntary or reflexive activity was seen, and visual threat showed a right homonymous hemianopia. Complete inferior bilateral face palsy was evidenced by Foix maneuver. Complete right arm and leg paralysis was noted and the patient was aphasic. NIHSS score was 22 points. The last dose of low-molecular-weight heparin was administered 13 hours before symptoms onset. A new head CT scan showed no significant changes when compared with the one performed 24 hours after first IV rtPA treatment (Figure 1(c)).

A thorough and thoughtful discussion regarding the appropriateness of the second IV thrombolytic treatment was held with the patient's family. Intra-arterial thrombolysis could have been an off-label alternative, but there was no availability at that moment. A second full-dose (0.9 mg/Kg) IV rtPA therapy was started 4 hours after stroke onset. Immediate NIHSS score after treatment was 18. Forty-eight hours later, NIHSS score was 16. The patient persisted with mild-to-moderate right hemiparesis, moderate aphasia, right homonymous hemianopia, and dysphagia. Head CT scan performed 24 and 48 hours (Figure 1(d)) after treatment showed the previous right lenticular infarction and a new predominantly subcortical ischemic lesion in the anterior region of the left middle cerebral artery territory with a PH2 (>30% of the infarcted area with space occupying effect) [11] hemorrhage. A pneumonia was diagnosed on day 20 and the patient died 5 days later. Death was attributed to the latter infectious complication.

We report a case of repeated IV rtPA within less than 5 days in a patient with paroxysmal atrial fibrillation and recurrent acute ischemic stroke. To the best of our knowledge, our case represents the third report on repeated systemic thrombolysis within 5 days between the incident and the recurrent ischemic stroke [6, 7] and the second one reporting on repeated IV rtPA treatment in a patient with early recurrent ischemic stroke associated to atrial fibrillation [7]. The current case raises several relevant issues related to repeated use of IV rtPA that warrant discussion: the potential development of anaphylactic reactions, the risk of hemorrhagic transformation, and the role of atrial fibrillation as a marker of early recurrent ischemic stroke and poor outcome after first and repeated IV thrombolysis.

There are few reports regarding potential anaphylactic reactions triggered by repeated thrombolysis [9]. Animal models have demonstrated the formation of antibodies against rtPA [12, 13]. However, development of these antibodies in humans is unusual [14] and there are only occasional reports of IV rtPA-related anaphylactic reactions in “real-life” clinical practice [15]. Moreover, no anaphylactic reactions have been described among any of the 13 cases of repeated IV thrombolysis for ischemic stroke reported up to date (including ours).

Our patient showed a PH2 hemorrhage on follow-up head CT scans which may have influenced the unfavorable neurological outcome, though this is controversial. Despite the short alpha half-life of rtPA (e.g., 4 to 5 minutes), its repeated use over short periods is contraindicated because of the risk of major bleeding, mainly intracerebral hemorrhage. In our patient, the risk of rtPA-associated hemorrhage was high because of the severity of the second stroke [16], the cardioembolic mechanism of the cerebrovascular event (atrial fibrillation) [17], and her history of congestive heart failure [18]. Damage to cerebrovascular walls due to free radical generation during reperfusion injury [19], the production matrix of metalloproteinases [20], and high concentrations of cellular fibronectin [21] have been implicated in the pathophysiology of rtPA-associated hemorrhage. However, the role of these biomarkers needs to be clarified.

The recovery of our patient was good after the first cerebrovascular event but she remained severely disabled after the second one, possibly because of the combination of recanalization failure and the hemorrhagic transformation. A strong association has been found between atrial fibrillation and early recurrent ischemic stroke after treatment with IV rtPA [22]. This phenomenon may be related to the disintegration of a preexisting intra-atrial thrombus during thrombolysis. As mentioned before, the risk of cerebral bleeding is also higher among atrial fibrillation patients after IV rtPA [17]. However, adverse neurological outcomes in these patients seem to be related more to recurrent embolization than to hemorrhagic transformation [22].

In conclusion, evidence-based medicine is the concept of treating patients according to the best available evidence. However, for many rare diseases and other situations like the very early recurrence of cardioembolic stroke in a patient who has received rtPA, it is often almost impossible to develop any form of randomized controlled trial. As mentioned before, international guidelines recommend avoiding repeated thrombolysis for patients with recurrent ischemic stroke within 3 months of the first event. This recommendation is not founded on randomized clinical trials, but is rather held on for the sake of patients' safety. For medical situations where impending permanent disability is the most probable scenario, “out-of-the-box” treatments could be used after a thorough discussion with patients and their families. We are aware that our medical decision was not supported by current guidelines and that most of the complications of systemic thrombolysis occur when recommendations are not followed. For this reason, based on teaching purposes and hoping to raise awareness about this important issue, we decided to report our case with the aim of further contributing to increase the scarce available evidence suggesting that early repeated systemic thrombolysis for recurrent ischemic stroke should be withheld.
